# Clonal analyses of refractory testicular germ cell tumors

**DOI:** 10.1371/journal.pone.0213815

**Published:** 2019-03-14

**Authors:** Michael T. Barrett, Elzbieta Lenkiewicz, Smriti Malasi, Melissa Stanton, James Slack, Paul Andrews, Lance Pagliaro, Alan H. Bryce

**Affiliations:** 1 Division of Hematology and Oncology, Mayo Clinic, Phoenix, Arizona, United States of America; 2 Department of Research, Mayo Clinic, Scottsdale, Arizona, United States of America; 3 Division of Anatomic Pathology, Mayo Clinic, Scottsdale, Arizona, United States of America; 4 College of Medicine, Mayo Clinic, Phoenix, Arizona, United States of America; 5 College of Medicine, Mayo Clinic, Rochester, Minnesota, United States of America; 2nd medical school of Charles University, CZECH REPUBLIC

## Abstract

Testicular germ cell tumors (TGCTs) are unique amongst solid tumors in terms of the high cure rates using chemotherapy for metastatic disease. Nevertheless, TGCTs still kill approximately 400 men per year, at a median age of 30 years, in the United States. This young age of mortality dramatically amplifies the impact of these deaths for the patients and their often young families. Furthermore the high cure rate makes it difficult to conduct further clinical trials of non curable disease. TGCTs are characterized by a marked aneuploidy and the presence of gain of chromosomal region 12p. Genomic testing may offer the ability to identify potentially lethal TGCTs at the time of initial diagnosis. However sequencing based studies have shown a paucity of somatic mutations in TGCT genomes including those that drive refractory disease. Furthermore these studies may be limited by genetic heterogeneity in primary tumors and the evolution of sub populations during disease progression. Herein we applied a systematic approach combining DNA content flow cytometry, whole genome copy number and whole exome sequence analyses to interrogate tumor heterogeneity in primary and metastatic refractory TGCTs. We identified both known and novel somatic copy number aberrations (12p, *MDM2*, and *RHBDD1)* and mutations (*XRCC2*, *PIK3CA*, *RITA1*) including candidate markers for platinum resistance that were present in a primary tumor of mixed histology and that remained after tandem autologous stem cell transplant.

## Introduction

It is estimated that in 2018 there will be close to 9,000 new cases of adult male germ cell tumors in the USA, leading to over 400 deaths [1]. Although this represents 0.4% of all cancer diagnoses in the USA, the impact of TGCT deaths is amplified by the young age of the patients, with a median age of death of 30 years resulting in the average life years lost being the highest amongst adult malignancies at 35 years. This young age of mortality dramatically amplifies the impact of these deaths for the patients and their often young families [2]. When the lost present value of lifetime earnings (PVLE) or so called productivity cost of cancer is considered, TGCTs carry a cost/death of $1.25 million which is nearly 6X greater than the average cost for cancer death, and more than twice as much as the second most costly malignancy. Thus there is an unmet need to identify biomarkers of high risk disease in primary tumors. Adult male germ cell tumors are exquisitely sensitive to platinum-based therapy such that advanced disease still carries a cure rate of approximately 70% [3, 4]. This high cure rate with metastatic disease is unique amongst adult solid tumors and is based in part on the exquisite chemo sensitivity of TGCTs. However the high cure rate limits the design of new studies to investigate the genomic basis of refractory disease and to improve the cure rate in these relatively young patients.

TGCTs are classified as seminoma, teratoma, embryonal carcinoma, yolk sac carcinoma, or choriocarcinoma, and the histology of clinical TGCT samples is frequently mixed with multiple components within a single tumor mass [5]. Although teratomas are considered nonmalignant and treated surgically, the multiple histologies are all highly responsive to cisplatin based chemotherapy [6]. TGCTs are characterized by a low mutation rate, a marked aneuploidy, and universal gain of chromosome arm 12p [6–10]. Sequencing based studies have reported recurring mutations in oncogenes and tumor suppressors [8, 11–13]. However the frequencies of these individual lesions account for only a small fraction of TGCTs. Thus key clinical questions related to the presence of driver genes on chromosome 12p and the basis of chemoresistance remain to be elucidated. In this report we describe our combined approach of DNA content based flow cytometry, whole genome copy number, and whole exome sequencing analysis of a small well annotated cohort of refractory TGCT. Notably these include primary and metastatic tissues in patients with mixed histology tumors. Our approach exploits the aneuploid nature of TGCTs to interrogate the genomes of each tumor and provides a unique analysis of refractory TGCTs including the evolution post chemotherapy of metastatic disease.

## Results

### Genomic lesions in refractory TGCT

#### DNA content flow cytometry

We detected aneuploid populations in each of the 5 TGCT cases analyzed (Table 1). The ploidies of each tumor varied from 2.7N to tetraploid and were present in primary and metastatic lesions with different histologies. The tumor nature of each aneuploid fraction was confirmed by genomic analyses. The diploid fractions from each tumor were also profiled to confirm that they were genomically normal and thus provide a patient matched normal for somatic analyses of these archived samples (S1 Fig). Two of the 5 cases had two distinct aneuploid populations in their tumor tissues. In patient 1 we detected a 2.7N and a 3.2N population in different regions of distinct histologies within the primary testicular mass, only one of which (2.7N) was present in three regions of resected post-transplant lymph node metastatic disease. The second case, patient 3, had a ploidy in the metastatic lesion (2.8N) that was distinct from the ploidy present in two regions of the primary tumor (3.0N).

**Table 1:** Tissue histology and ploidy of Chemoresistant TGCTs.

Case	Origin	Primary	Metastasis
1	Transplant	3^a^ Seminoma (3.2N)	A1^a^ Teratoma (2.7N)
	Transplant	5 Mixed (Embryonal, YST^b^, Teratoma) (2.7N)	A2 Choriocarcinoma (2.7N)
	Transplant		A3 Choriocarcinoma (2.7N)
2	Recurrent	A7 Seminoma (4.0N)	
	Recurrent	A8 Embryonal (4.0N)	
3	Recurrent	A1 Mixed (3.0N)	A1 (2.8N)
	Recurrent	2 Mixed (3.0N)	
4	Transplant	A3 Choriocarcinoma (3.0N)	H2 Choriocarcinoma (3.2N)
	Transplant	A7 ITGCN^c^ (nd^d^)	H3 Choriocarcinoma (nd)
	Transplant	11 Seminoma + Sarcoma (3.0N)	
5	Transplant	Seminoma (3.0N)	

^a^Tissue block number

^b^YST: Yolk sac tumor

^c^ITGCN: Intratubular germ cell neoplasia

^d^nd: Not determined

#### 12p amplicon

**Fig 1:**
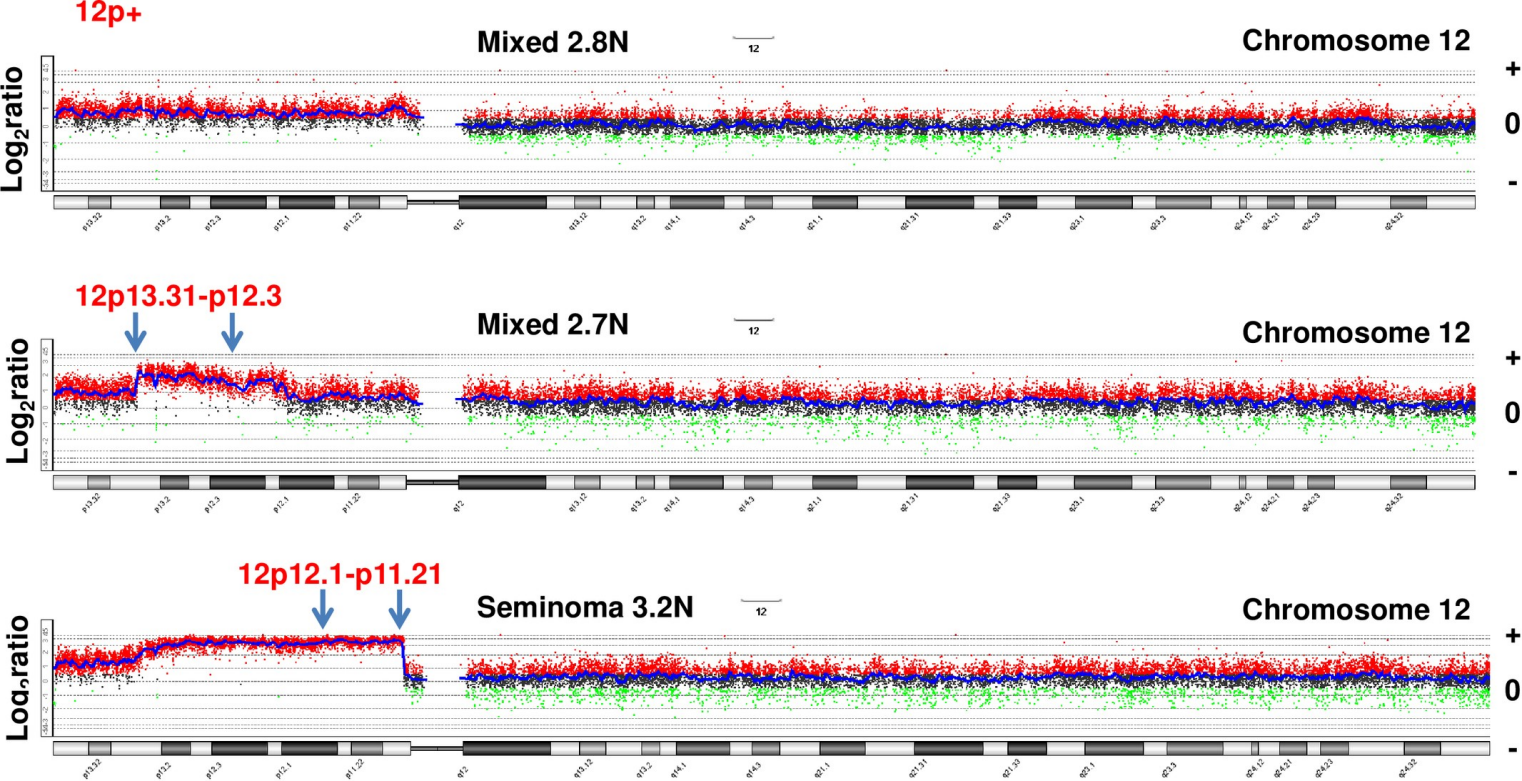
Mapping 12p amplicons in TGCT genomes. Copy number aberrations on chromosome 12p in three distinct TGCT aneuploid populations. The 12p amplicons included the whole 12p arm (3/5 cases) in a 2.8N population detected in case #3 and two distinct amplicons in the 2.7N and the 3.2N populations present in case #1. The X and Y axes in the CGH plots represent chromosome and log_2_ratios for each TGCT.

All five patients had gains of 12p in their aneuploid tumor genomes (Fig 1). In four cases (#2–5) the amplified region included the whole p arm of chromosome 12 (Table 2). Strikingly there were distinct patterns of 12p copy number gains in the two aneuploid TGCT populations present in patient 1. The maximum region of amplification (log_2_ratio >3.5) in the 3.2N genome extends from the *BCAT1* locus at p12.1 to the *FGD4* locus at p11.21 and includes *KRAS*. In contrast each of the 2.7N aneuploid populations within the primary and metastatic lesions shared a 12p amplicon that had a lower level of amplification (log_2_ratio >2.0) with a broader maximal region extending from *CLEC4C* at p13.31 to *DERA* at p12.3. The lack of overlap between these two regions of maximum amplification suggests that the 12p amplicons diverged during the evolution of the aneuploid lineages in the primary tumor. Notably the p13.31-p12.3 peak of the 2.7N amplicon is wholly contained within the lower amplified region adjacent to the 3.2N amplicon. However, the highest region of overlap between the 2.7N and 3.2N amplicons spanning p13.31-p12.1 includes candidate TGCT 12p driver genes *NANOG*, *ETV6*, and *ATF7IP* [9, 14, 15].

**Table 2:** Copy number variants.

Case-Ploidy	CNV	Log_2_Ratio	Interval Genes
1–2.7N	12p13.31—p12.3	2.1	*NANOG*, *ETV6*, *ATF7IP*
	4q11—q21.1	0.7	*KIT*
	7p	0.6	
	13q13.2—q34	-1.0	
	11q14.1—q25	-0.7	
1–3.2N	12p12.1—p11.21	3.5	*NANOG*, *ETV6*, *ATF7IP*, *KRAS*
	4q12	2.3	*KIT*
	7p22.3—p14.1	0.9	
	11q21—q24	-1.1	
	18	-0.5	
	13q	-0.5	
	3	-0.5	
2–4.0N	12p	0.9	
	18q11.2—q12.1	0.5	
3–2.8N	12p	0.8	
	13q14.3—q34	-0.3	
4–4.0N	12p	0.4	
5–3.0N	12q15	3.9	*MDM2*
	2q36.3	3.7	*IRS2*, *RHBDD1*
	6p22.2	1.9	*HIST1* cluster
	12p	1.0	
	1q	0.7	
	21q	0.6	
	3q26.32—q27.3	0.6	*PIK3CA*^*a*^
	7	0.5	
	3q27.3—q29	-0.8	*BCL6*, *TP63*
	4q	-0.7	
	13q	-0.4	
	11	-0.4	
	18q	-0.4	

^a^ W1057*

#### Focal amplicons in TGCT genomes

**Fig 2:**
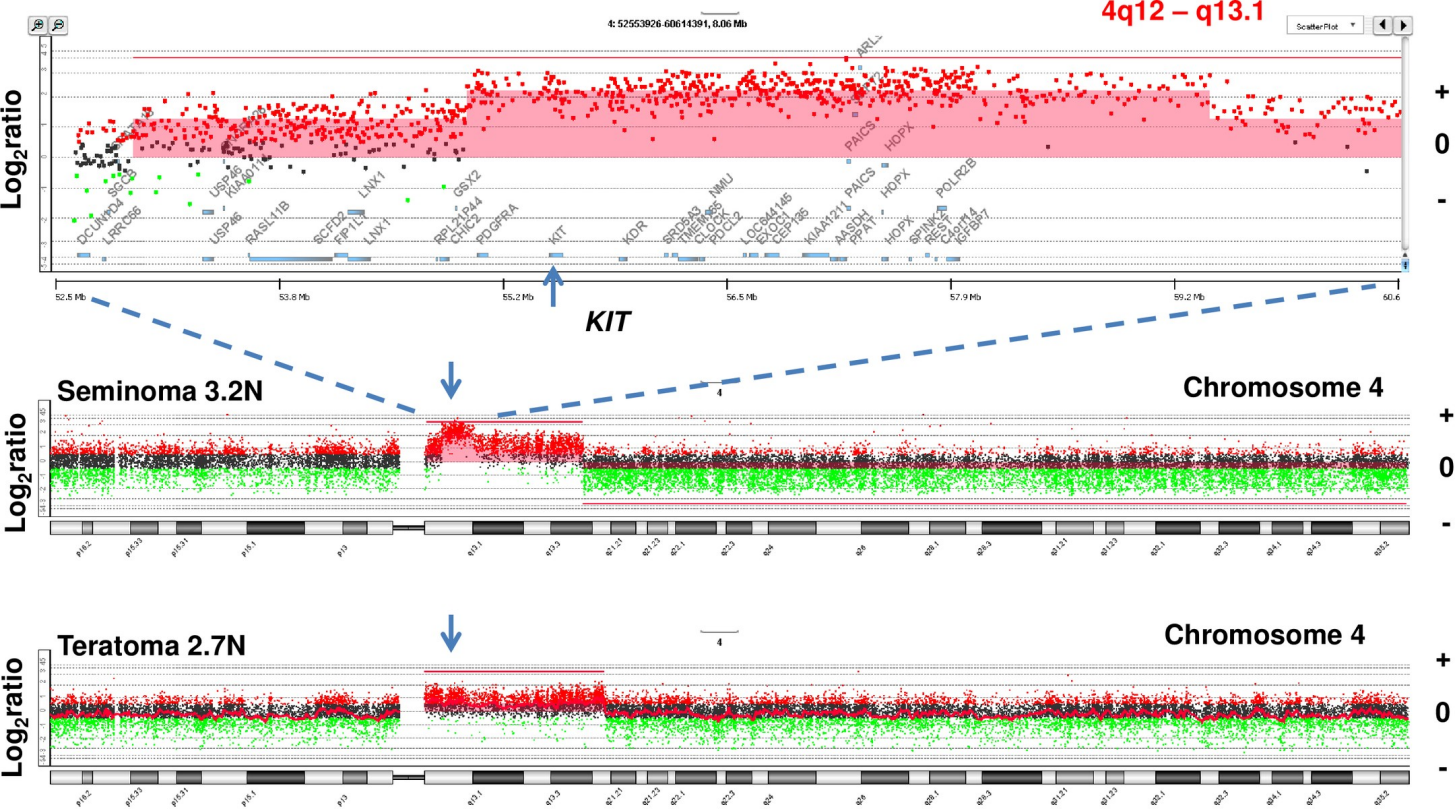
Mapping 4q amplicons targeting *c-KIT* oncogene. Copy number aberrations targeting 4q in case #1. The red shaded areas denote ADM2 defined copy number aberrant intervals. The X and Y axes in the CGH plots represent chromosome and log_2_ratios for each TGCT.

**Fig 3:**
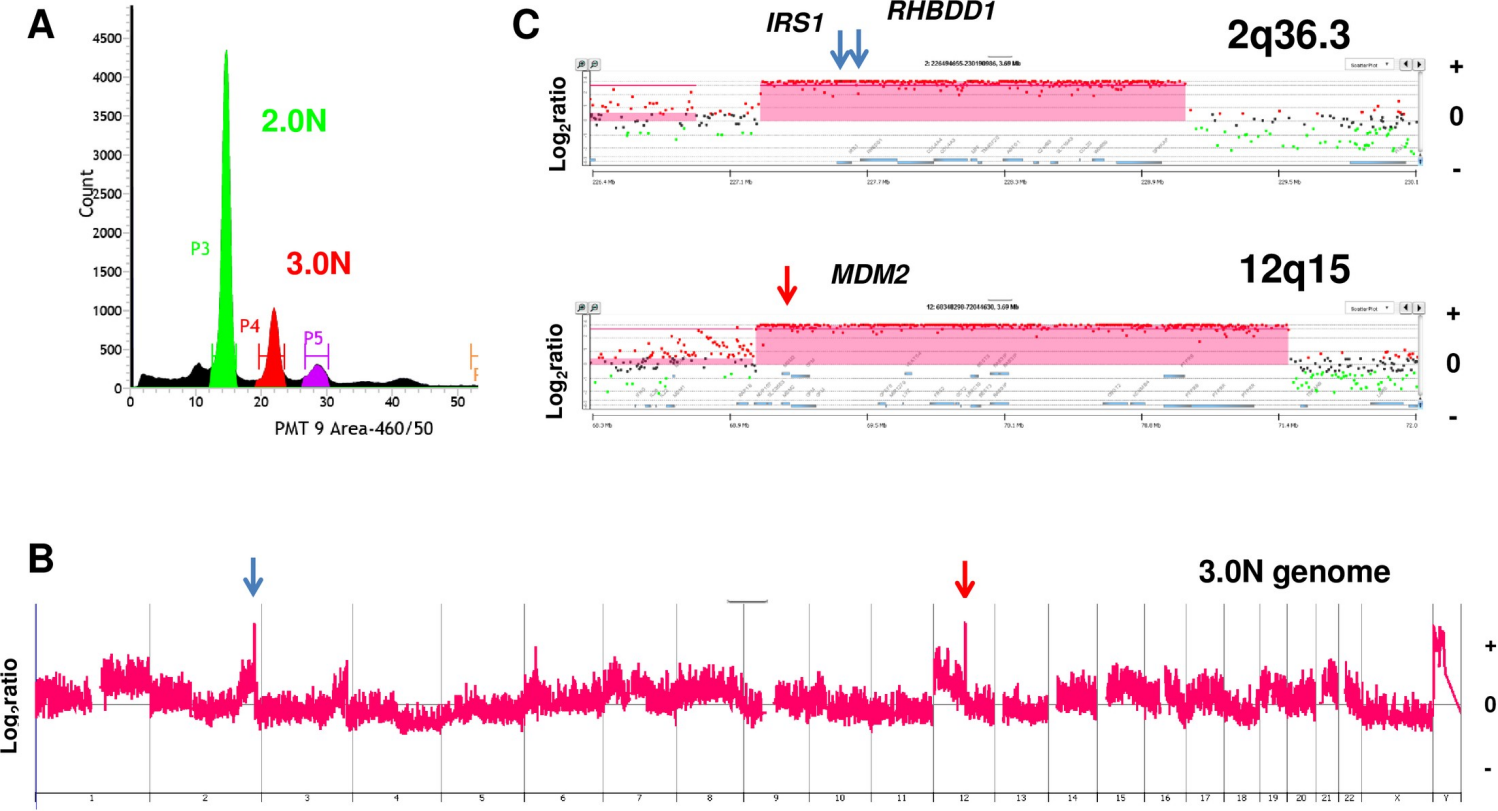
Clonal analysis of refractory metastatic TGCT. A) DNA content flow sorting of aneuploid (3.0N) and diploid (2.0N) populations from primary FFPE tissues for case #5. B) Whole genome copy number plots for 3.0N population included 12q15 and 2q 36.3 amplicons targeting *MDM2* (red arrow) and both *IRS2* and *RHBDD1* (blue arrows) in each aneuploid genome. C) Locus specific view of each amplicon. The X and Y axes in the CGH plots represent chromosome and log_2_ratios. Red shaded areas denote ADM2 defined CNV interval.

In addition to their distinct 12p amplicons the two aneuploid populations in patient 1 had overlapping 4q amplicons that included the *KIT* locus (4q12), a known oncogenic driver in TGCTs (Fig 2). Notably the 3.2N population had a region of increased copy number gain internal to the region of overlap that included *KIT* suggesting ongoing selection during the clinical history of the tumor. We detected three additional focal amplicons in one of the remaining refractory cases. These included a high level (log_2_ratio >3.5) amplicon targeting *MDM2* (12q15) and another targeting both Insulin Receptor Substrate 1 (*IRS1*) and Serine Protease Rhomboid Domain Containing 1 (*RHBDD1*) (2q36.3) in the 3.0N population present in the seminoma tissue of patient 5 (Fig 3). *RHBDD1* is highly expressed in testis and promotes apoptosis during normal spermatogonia development [16, 17]. To our knowledge this amplicon has not previously been described in TGCTs. Given the low frequency of copy number variants (CNVs) in these tumor genomes, the height and the focal nature of the *MDM2* and the 2q36.3 amplicon suggest they were highly selected during the clinical history of this refractory TGCT. The third focal amplicon in this population targeted the histone cluster on 6p22.2 (S2 Fig).

In addition to these amplicons there were a series of low level copy number gains and losses targeting broad regions of tumor genomes in individual cases (Table 2). However the only recurring events were losses on 13q in 4/5 patients with a large region of overlap extending from q14.3 to q34, and gain of the entire p arm of chromosome 7 in 2/5 patients.

#### Somatic nucleotide variants

We sequenced the exomes of 11 flow sorted tumor populations and matching sorted non tumor diploid populations from the 5 patients (Table 3). Mutation frequencies were consistent with previous reports of TGCTs and all 5 cases were *TP53* and *KRAS* wild type [8, 11, 12, 15]. However we detected individual somatic variants in known oncogenes (e.g. *PIK3CA*) and tumor suppressors (e.g. *CDC20*). Notably, the *PIK3CA*^w1057^* mutation was present in a 3.0N genome with a gain of chromosome 3q26.32 –q27.3 in patient 5 and has been reported as a pathogenic somatic variant in multiple tumors [18] (S3 Fig). In addition we detected a *NRAS*^C118Y^ variant in case #2. This rare mutation is predicted to constitutively activate Ras-GTP and lead to hyperactive Ras signaling [19].

**Table 3:** Targeted somatic lesions in 12p+ refractory TGCTs.

Case	DNA Repair	Apoptosis	Oncogenic Signaling
1	XRCC2^R188H^		KIT amplicon
2			NRAS^C118Y^
3	XRCC2^D34Y^		
4	XRCC2^D82Y^		
5	MDM2 amplicon	RHBDD1 amplicon	PIK3CA^W1057^*

**Fig 4:**
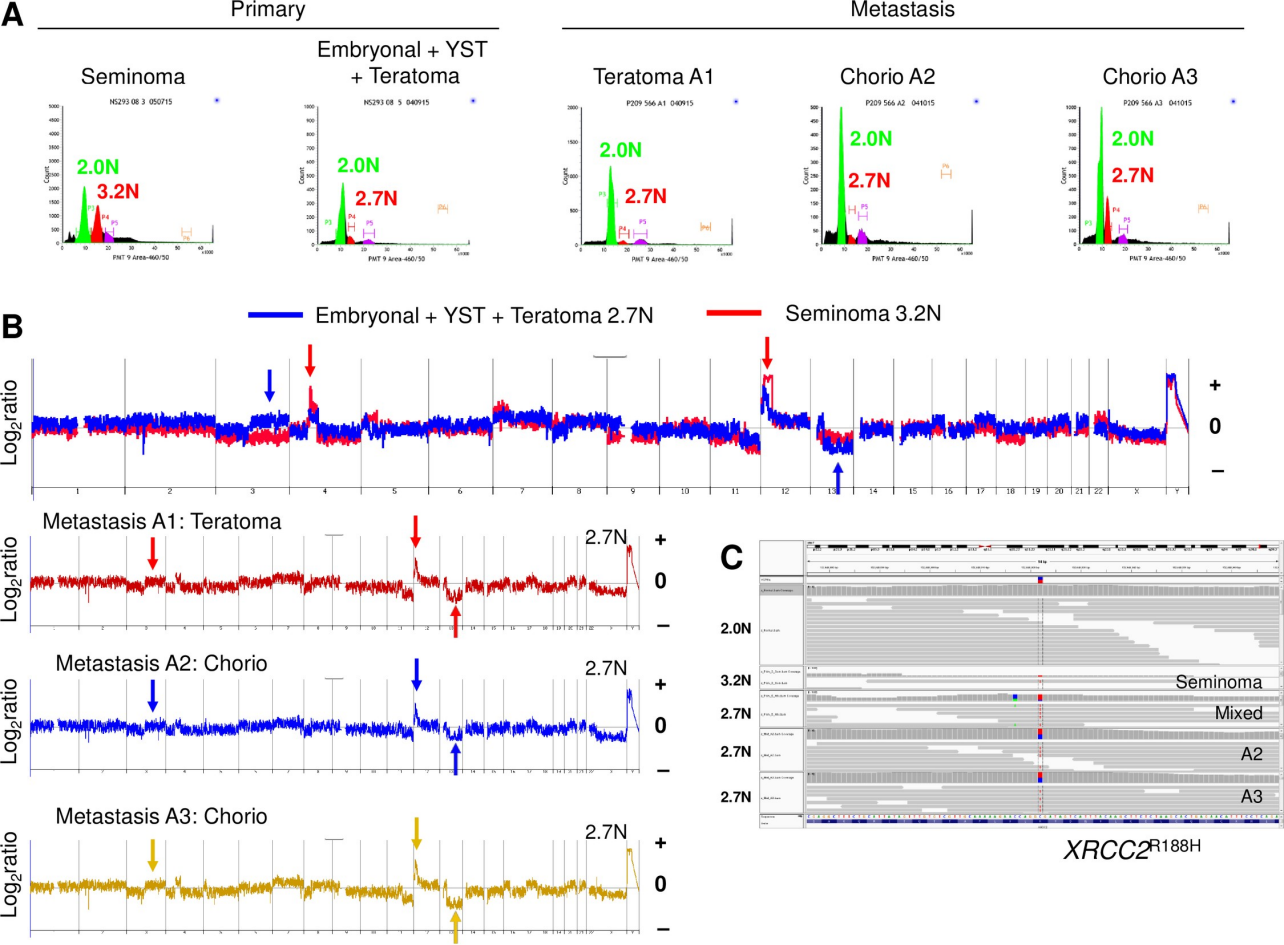
Clonal analysis of primary and refractory metastatic TGCT. A) DNA content flow sorting of aneuploid (3.2N, 2.7N) and diploid (2.0N) populations from primary and metastatic FFPE tissues of case #1. The histology of each biopsy is listed above the histogram. B) Copy number aberrations included 4p amplicons targeting *c-KIT* (red arrow) and distinct 12p amplicons (red and blue arrows) in each aneuploid genome. In addition a gain of 3q and deletion of 13q13.3-qtel were unique to the 2.7N genomes (blue arrows). C) IGV view of the clonal *XRCC2*^R188H^ mutation in each aneuploid population and the wild type sequence in patient matched normal 2.0N population.

We also detected mutations in *XRCC2* in 3/5 patients with refractory disease. In case 1 each tumor population shared a clonal *XRCC2*^R188H^ mutation, regardless of ploidy, 12p amplicon, histology and anatomical site (Fig 4) [20]. In contrast the diploid (2.0N) populations from each biopsy were copy number neutral and wild type for variants including *XRCC2*^R188H^ confirming the somatic nature of the genomic aberrations. Cases 3 and 4 had additional non conserved somatic *XRCC2* mutations. In the former the mutation was present in a 2.8N population present in a metastatic lesion but absent from a 3.0N population present in two primary lesions. In the second case the *XRCC2* variant was detected in the seminoma portion of the primary tumor but absent from the choriocarcinoma. In both cases the VAFs were lower than in case 1 suggesting either a sub clonal *XRCC2* wild type population within the tumors or admixtures of non tumor cells in the sorted samples.

In addition to these likely pathogenic variants we also detected somatic variants within known cancer genes including *BUB1*, *MSH2*, and *KIT*. However in each example the variants occurred outside known hot spots and critical protein domains, involved conserved amino acid changes, or have been predicted to be non-pathogenic [21, 22].

#### Clonal evolution of chemorefractory TGCT

We used the combined ploidy, CNV and mutation data from patient 1 to recreate the cell lineage that evolved during the clinical history of the disease and define the tumor population(s) that was present in the refractory metastatic lesions. The patient was initially diagnosed at 28 years old when he presented with a right testicular mass. Orchiectomy demonstrated a mixed non seminomatous germ cell tumor (NSGCT) consisting of 50% seminoma, 30% embryonal carcinoma, and 20% teratoma. Preoperative α-fetoprotein (AFP), β- human chorionic gonadotropin (βHCG), and lactate dehydrogenase (LDH) were normal and the abdominal computerized tomography (CT) scan demonstrated retroperitoneal lymphadenopathy. Two months after the orchiectomy the patient underwent a retroperitoneal lymph node dissection (RPLND) which demonstrated mixed NSGCT in 8 of 19 resected lymph nodes with a predominance of mature and immature teratoma, choriocarcinoma, and small foci of embryonal and yolk sac carcinomas. The patient then began surveillance.

Three months after surgery, βHCG rose to 361 and a CT scan of the abdomen demonstrated recurrent retroperitoneal lymphadenopathy up to 2.2 cm. Left testicular ultrasound was normal and the patient began chemotherapy with bleomycin, etoposide and cisplatin (BEP) for 3 cycles. βHCG levels fell to 26 after BEP, but the following month βHCG levels rose again to 214. Now eight months after initial diagnosis, repeat imaging demonstrated further progressive lymphadenopathy up to 3.8 cm in the abdomen with no disease in the chest or central nervous system (CNS). The patient was referred to our institution for consideration of high dose chemotherapy and autologous stem cell transplant (ASCT) [23, 24]. He underwent tandem autologous stem cell transplant with carboplatin and etoposide. After cycle 2 his βHCG fell from 450 pre transplant to 12. Pretreatment imaging showed that the lymphadenopathy increased to 4.5cm which reduced to only 4.3 cm post-transplant. One month later the βHCG had risen to 62 and the patient was taken for resection of all residual disease. Pathology demonstrated residual choriocarcinoma and teratoma forming a 3 cm mass with extensive necrosis. The βHCG subsequently fell to <0.2 and the patient was alive with no residual disease after 5 ½ years of follow up.

**Fig 5:**
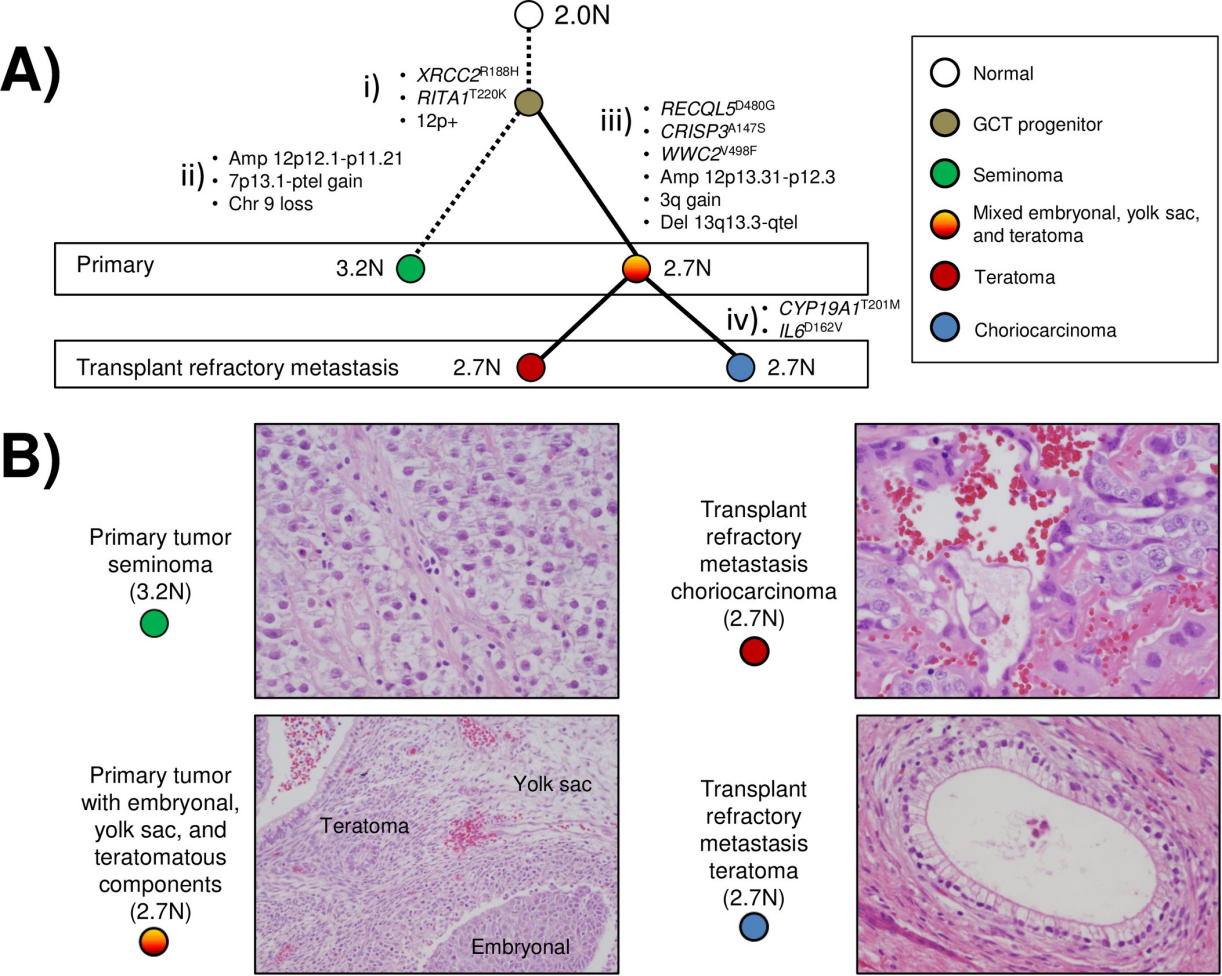
Cell lineage of metastatic TGCT. A) Genomic aberrations within primary and refractory metastatic TGCT in case #1 include; i) shared *XRCC2*^*R188H*^ and *RITA1*^T220K^ mutations in an inferred malignant progenitor; ii) CNVs, 12p13.31-p11.21 amplicon, gain of 7p13.1-ptel, and loss of chromosome 9, that are private to the primary 3.2N tumor seminoma population; iii) mutations *RECQL5*^D480G^, *CRISP3*^A147S^, *WWC2*^V498F^, and CNVs 12p13.31-p12.3, gain of 3q, and deletions of 13q13.3-qtel, private to the 2.7N population within the non-seminomatous primary and metastatic tumor; iv) mutations *CYP19A*1^T201M^, and *IL6*^D162V^, that are private to 2.7N metastatic population. B) Haematoxylin and eosin (H&E) staining and visualization of distinct TGCT histologies within the primary and metastatic tumor tissues.

Within the primary tumor a region of seminomatous disease contained an aneuploid 3.2N population while the region of mixed embryonal, yolk sack, and teratoma histology had a distinct 2.7N population (Fig 4A). The transplant refractory metastases consisted of teratoma and choriocarcinoma, from which we sorted and analyzed one region of teratoma and two regions of choriocarcinoma. All three of these samples had the same 2.7N ploidy as the non seminomatous components from the primary tumor with no evidence of the 3.2N population. We simultaneously isolated co-existing 2.0N diploid populations from each sample and processed them in parallel with the aneuploid population. Each tumor population had a shared clonal *XRCC2*^R188H^ mutation, regardless of ploidy, 12p amplicon, histology and anatomical site (Fig 4B and 4C) [20]. In contrast the diploid (2.0N) populations from each biopsy were copy number neutral and wild type for variants including *XRCC2*^R188H^ confirming the somatic nature of the genomic aberrations. The genomic lesions among the aneuploid tumor populations defined the metastatic TGCT cell lineage (Fig 5). These included private CNVs within the primary non-seminomatous component, CNVs and mutations shared within the seminomatous component, and mutations that were private to the transplant refractory metastasis.

## Discussion

An emerging picture points to cancer as an evolutionary process of branched clonal evolution, in which molecular characteristics of individual populations within each “branch” or lineage determine a tumor’s ability to progress, respond to therapy, and develop resistance [25–28]. Thus clonal mutation(s) present within a primary tumor can drive tumor evolution and affect the clinical course of disease. We have advanced the application of solid tissue flow cytometry to identify and purify the nuclei of tumor populations directly from clinical tissues for subsequent whole genome and exome analyses [29–33]. Significantly for this study it is well established that TGCTs are aneuploid [7, 34]. Thus DNA content based sorting of each GCT sample of interest provides enriched tumor populations for analysis and a matching genomically normal diploid population for distinguishing germ line and somatic events. In this current dataset we have analyzed 17 pre and post chemotherapy tissues from five men with fatal TGCT. These data consists of ploidy, whole genome copy number, and whole exome mutation analysis for each refractory TGCT patient.

The recurring nature of the 12p amplicon supports the role of one or more genes in this region as drivers of TGCT [6]. The 12p amplicon mapping identified a core region that included candidate driver genes *NANOG*, *ETV6* and *ATF7IP*. This region was conserved in the 2.7N population that was present in primary and metastatic tissues in patient 1. In contrast *KRAS* was included in the higher focal 12p amplicon of the 3.2N population that was only detected in the primary seminoma tissue. Furthermore we did not detect any mutations in *KRAS* in any of the sorted tumor populations from each patient. These results suggest that although *KRAS* mutations have been detected in TGCTs and the *KRAS* locus is frequently included in the 12p amplicon it did not drive refractory TGCT in the current cases. In addition the frequencies of amplification and mutations targeting *KIT* have established its role as a key oncogene in the development of disease. The inclusion of *KIT* in the SRO between the 4q amplicons in the 3.2N and 2.7N populations in case 1 supports a role for *KIT* activation in the early stages of TGCT progression. Alternatively relatively rare but high level focal amplicons also represent selected lesions in genomes of interest. Given the paucity of genomic aberrations in TGCT genomes the presence of distinct high level amplicons provides candidate genes for the evolution of refractory disease.

The role of clonal evolution in refractory TGCT is highlighted in case 1. We were able to interrogate the two regions of the primary testicular mass and the three regions of resected post-transplant lymph node metastatic disease with our solid tissue flow cytometry based methods. Tissue from the pre transplant RPLND could not be obtained from the outside institution. The presence of a clonal *XRCC2*^R188H^ mutation in each sorted tumor population highlights the role of clonal evolution in refractory disease. Our results suggest that pre-existing genomic lesions targeting DNA repair and apoptosis provide selective mechanisms for the evolution of refractory TGCT. Mutations in *XRCC2* have recently been reported in 2 isolated cases of refractory TGCTs [8]. However the prognostic significance of these mutations remains to be determined. The clonal somatic *XRCC2*^R188H^ mutation we detected in this patient is a variant that has been shown to confer resistance to cisplatin induced DNA damage in cell based models [20]. In this case the *XRCC2*^*R188H*^ and a *RITA1*^T220K^ mutation appear to be early somatic events occurring prior to the development of the distinct 12p amplicons. The double mutant progenitor cell then branched into two separate developmental pathways; one leading to the seminoma component which acquired a series or private CNVs and mutations, and a second leading to the non seminomatous component with a more restricted set of private CNVs (Figs 4 and 5). Given this pattern of somatic lesions and the presence of *XRCC2* mutations in 2 additional refractory cases we propose that clonal mutations in *XRCC2* and most likely in other related RecA/Rad51 homologous DNA repair genes are present in a subset of TGCTs prior to therapy and promote the clonal selection of refractory disease.

In addition to *XRCC2* mutations the presence of high level focal amplicons targeting *MDM2* and both *IRS2* and *RHBDD1* suggest that these events can be highly selected in the evolution of refractory TGCT. *MDM2* amplicons have been reported previously in TGCTs and were associated with cisplatin resistance and poorer outcomes ([8, 35, 36]. Given the absence of *TP53* mutations in our selected cohort increased *MDM2* likely provides a highly selected mechanism to abrogate cell cycle checkpoints and DNA repair during the evolution of disease. Amplification of *IRS1* has been previously reported in a case of cisplatin resistant TGCT [36]. However this was detected using a targeted gene panel sequencing approach that did not include mapping of the 2q36.3 amplicon and the inclusion of *RHBDD1* as a co-amplified gene. *RHBDD1* is highly expressed in the testis and is involved in the negative regulation of apoptosis of spermatogonia during normal development [16, 17]. The co-occurrence of these two high level focal amplicons in the same tumor genome highlight the potential role of apoptosis in refractory TGCTs. Notably recent reports have described high mitochondrial priming as a mechanism of chemotherapy-induced apoptosis that promotes chemo sensitivity [15]. Thus the co-occurrence of the *MDM2* and *RHBDD1* amplicons may provide a key driver of resistance in this case.

Ultimately investigators need to be able to evaluate whether more aggressive first line treatment of patients with markers of resistance and relapse would increase the cure rate or whether novel therapies will be required. Consequently a goal of our ongoing investigations into the clonal basis of refractory TGCT is to develop an efficient biomarker panel to address this clinical need. Our preliminary results in this small cohort have identified potential mediators of refractory TGCT (Table 3). These include *XRCC2* mutations and focal amplicons targeting known (*MDM2*) and novel candidate oncogenes (*RHBDD1*) in refractory TGCT. These results, notably in paired primary and metastatic tissues acquired from single patients during the clinical history of their refractory disease highlight how our flow sorting based clonal methods provide a unique precision genomics approach to the study of TGCTs.

## Materials and methods

### Clinical samples

This study was approved by the Mayo Clinic Institutional Review Board (IRB). Clinical information and formalin fixed paraffin embedded (FFPE) tissue were gathered from the patient records at Mayo Clinic under IRB protocol number 13–003123. All study conduct was done in accordance with the principles expressed in the Declarations of Helsinki (https://www.wma.net/policies-post/wma-declaration-of-helsinki-ethical-principles-for-medical-research-involving-human-subjects/).

### Flow sorting FFPE tissues

Nuclei from diploid, tetraploid, and aneuploid cell populations present in each FFPE TGCT tissue were sorted using our published protocols [31, 37]. Briefly, excess paraffin is removed with a scalpel from individual 40-60um scrolls which are then washed with 1ml Xylene for 5 minutes. The samples are filtered through a 35um mesh and resuspended in a final concentration of 10ug/ml DAPI prior to flow sorting with an Influx cytometer with ultraviolet excitation (Becton-Dickinson, San Jose, CA). DNA content and cell cycle are then analyzed using MultiCycle (Phoenix Flow Systems, San Diego, CA).

### DNA extraction

DNA from sorted nuclei was extracted using an amended protocol from QIAamp DNA Micro Kit from Qiagen (Valencia, CA). Briefly each sorted sample was resuspended in 180μl buffer ATL and 20μl proteinase K then incubated for 3 hours at 56°C for complete lysis. Samples were bound and washed according to QIAamp DNA Micro Kit instructions, eluted into 50μl of H_2_0, then precipitated overnight with 5μl 3 M sodium acetate and 180 μl 100% EtOH. Each sample was then centrifuged for 30 minutes at 20,000 x g, washed in 1 ml of 70% EtOH for 30 minutes at 20,000 x g. The samples were carefully decanted and the DNA pellet was dried by speed vacuum then resuspended in a small volume (e.g. 10–50μl) of H_2_0 for final concentrations suitable for accurate quantification.

### DNA amplification

Genomic DNAs from sorted FFPE samples were amplified using the Ovation WGA FFPE System from NuGEN Technologies (San Carlos, CA). For the latter samples (patients #1–3) DNA was processed in accordance with Ovation WGA FFPE standard SPIA protocol with an alternate T7 endonuclease fragmentation step. Resulting amplified product was either used as template for aCGH analysis or processed with the NuGEN Encore ds-DNA module according to the supplier’s instructions in order to generate double-stranded (ds) end repaired DNA as input for libraries suitable for next generation sequencing (NGS). A 100 ng aliquot of pooled 46,XX DNA (Promega, Madison, WI) was amplified with the matching amplification protocol to generate a suitable reference for each NGS and aCGH experiment. In all cases the quality of the amplification product was assessed by gel electrophoresis. In two cases (patients 4 and 5) we used the Rubicon ThruPLEX DNA-seq kit (Ann Arbor, MI) on extracted genomic DNA to prepare additional templates for whole exome sequencing.

### aCGH analysis

Sample and reference templates were labeled with Cy-5 dUTP and Cy-3 dUTP respectively using a BioPrime labeling kit (Invitrogen, Carlsbad, CA) according to our published protocols[30, 37]. All labeling reactions were assessed using a Nanodrop assay (Nanodrop, Wilmington, DE) prior to mixing and hybridization to 400k CGH arrays (Agilent Technologies, Santa Clara, CA) for 40 hours in a rotating 65°C oven. All microarray slides were scanned using an Agilent 2565C DNA scanner and the images were analyzed with Agilent Feature Extraction version 11.0 using default settings. The aCGH data was assessed with a series of QC metrics then analyzed using an aberration detection algorithm (ADM2)[38]. The latter identifies all aberrant intervals in a given sample with consistently high or low log ratios based on the statistical score derived from the average normalized log ratios of all probes in the genomic interval multiplied by the square root of the number of these probes. This score represents the deviation of the average of the normalized log ratios from its expected value of zero and is proportional to the height h (absolute average log ratio) of the genomic interval, and to the square root of the number of probes in the interval. All aCGH data discussed in this publication have been deposited in NCBI's Gene Expression Omnibus [39] and are accessible through GEO Series accession number GSE123464 https://www.ncbi.nlm.nih.gov/geo/query/acc.cgi?acc=GSE123464.

### Whole exome sequencing

DNAs from each sorted tumor population and a patient matched control sample were sequenced within the Mayo Clinic Medical Genome Facility (MGF) using established protocols for whole exome analysis. Briefly, whole exon capture was carried out with Agilent’s SureSelect Human All Exon 71 MB v6 kit. 500 ng of the prepped library is incubated with whole exon biotinylated RNA capture baits supplied in the kit for 24 hours at 65°C. The captured DNA:RNA hybrids are recovered using Dynabeads MyOne Streptavidin T1 (Dynal). The DNA was eluted from the beads and desalted using purified using Ampure XP beads (Agencourt).The purified capture products were then amplified using the SureSelect Post-Capture Indexing forward and Index PCR reverse primers (Agilent) for 12 cycles. Libraries were loaded onto paired end flow cells at concentrations of 4–5 pM to generate cluster densities of 600,000–800,000/mm^2^ using the Illumina cBot and HiSeq Paired end cluster kit version 3.The flow cells are sequenced as 101 X 2 paired end reads on an Illumina HiSeq 2500 or 4000 using TruSeq SBS sequencing kit version 3 and HiSeq data collection version 1.4.8 software. Base-calling was performed using Illumina’s RTA version 1.12.4.2.

## Supporting information

S1 Fig Flow sorting and CNV profiles of diploid (2.0N) and aneuploid (3.0N) populations from a TGCT biopsy. DNA content analysis of diploid and aneuploid populations flow sorted from FFPE TGCT tissue. The X and Y axes in the CGH plots represent chromosome and log_2_ratios.
(TIF)

S2 Fig Histone H1 cluster amplicon. Whole genome (bottom panel) and locus-specific (top panel) view of focal 6p22 amplicon targeting the Histone cluster in case #5. The red shaded areas denote ADM2 defined copy number aberrant intervals. The X and Y axes in the CGH plots represent chromosome and log_2_ratios.
(TIF)

S3 Fig Somatic lesions targeting PIK3CA in aneuploid TGCT population. Whole genome (bottom panel) and chromosome 3 (middle panel) CNV profiles. The red shaded areas denote ADM2 defined copy number aberrant intervals. The X and Y axes in the CGH plots represent chromosome and log_2_ratios. C) IGV view of somatic *PIK3CA* mutation (top panel).
(TIF)
