# Supplementary figures and images for: Clonal analyses of refractory testicular germ cell tumors
Source: PLoS One. 2019 Mar 14;14(3):e0213815. doi: 10.1371/journal.pone.0213815 (PMC6417677; doi:10.1371/journal.pone.0213815)

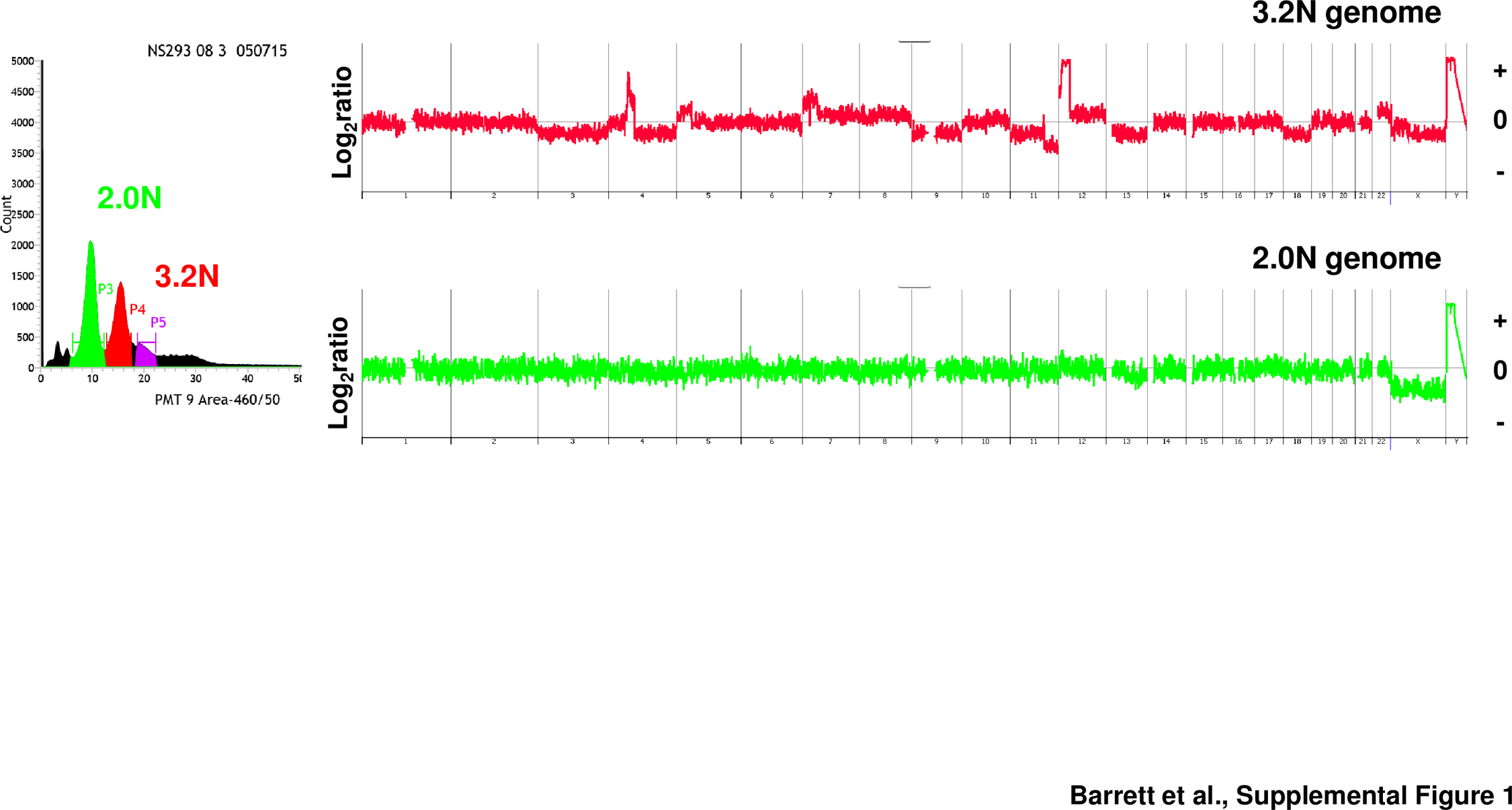

Supplement: S1 Fig — DNA content analysis of diploid and aneuploid populations flow sorted from FFPE TGCT tissue. The X and Y axes in the CGH plots represent chromosome and log2ratios. (TIF) [file pone.0213815.s001.tif]

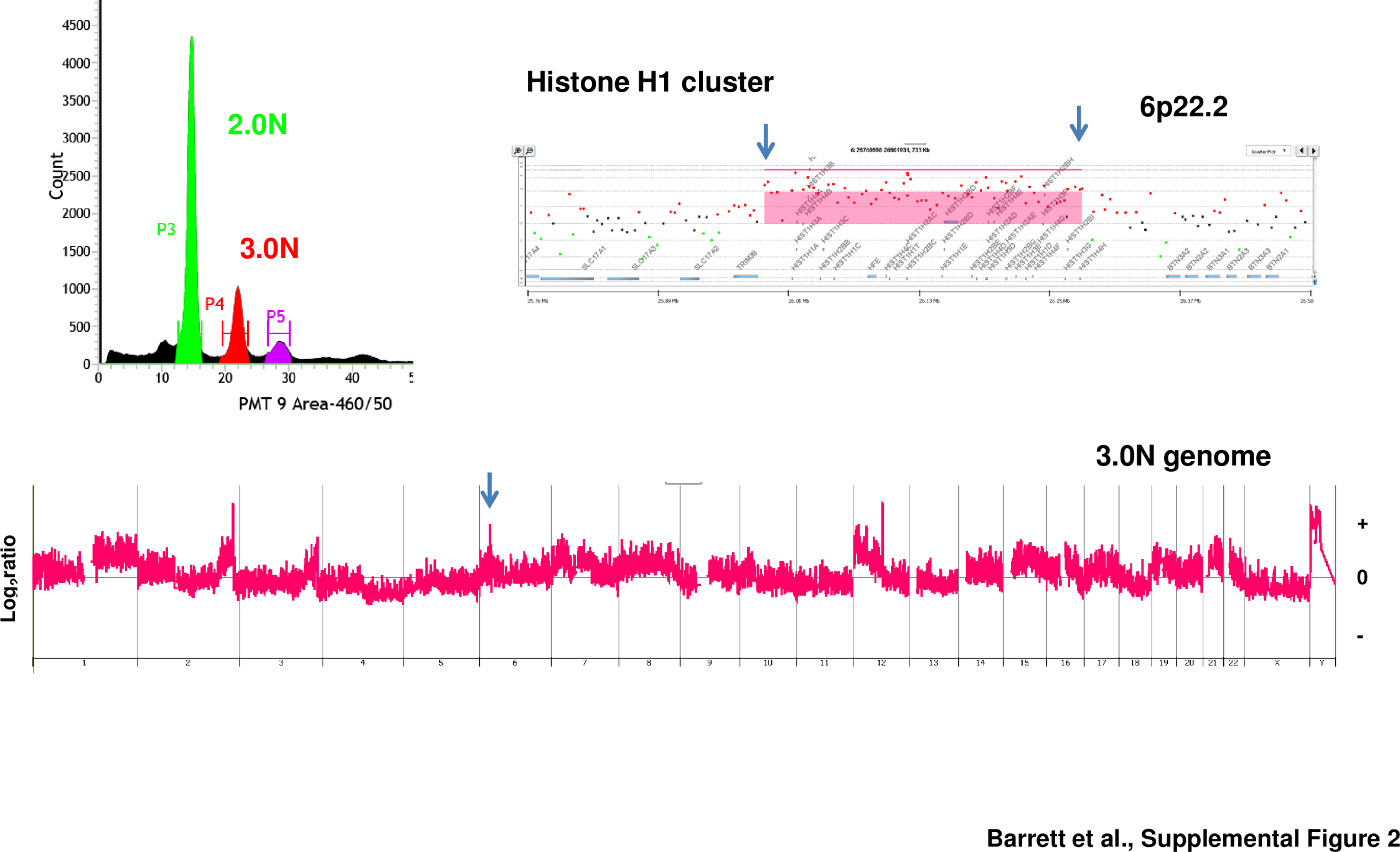

Supplement: S2 Fig — Whole genome (bottom panel) and locus-specific (top panel) view of focal 6p22 amplicon targeting the Histone cluster in case #5. The red shaded areas denote ADM2 defined copy number aberrant intervals. The X and Y axes in the CGH plots represent chromosome and log2ratios. (TIF) [file pone.0213815.s002.tif]

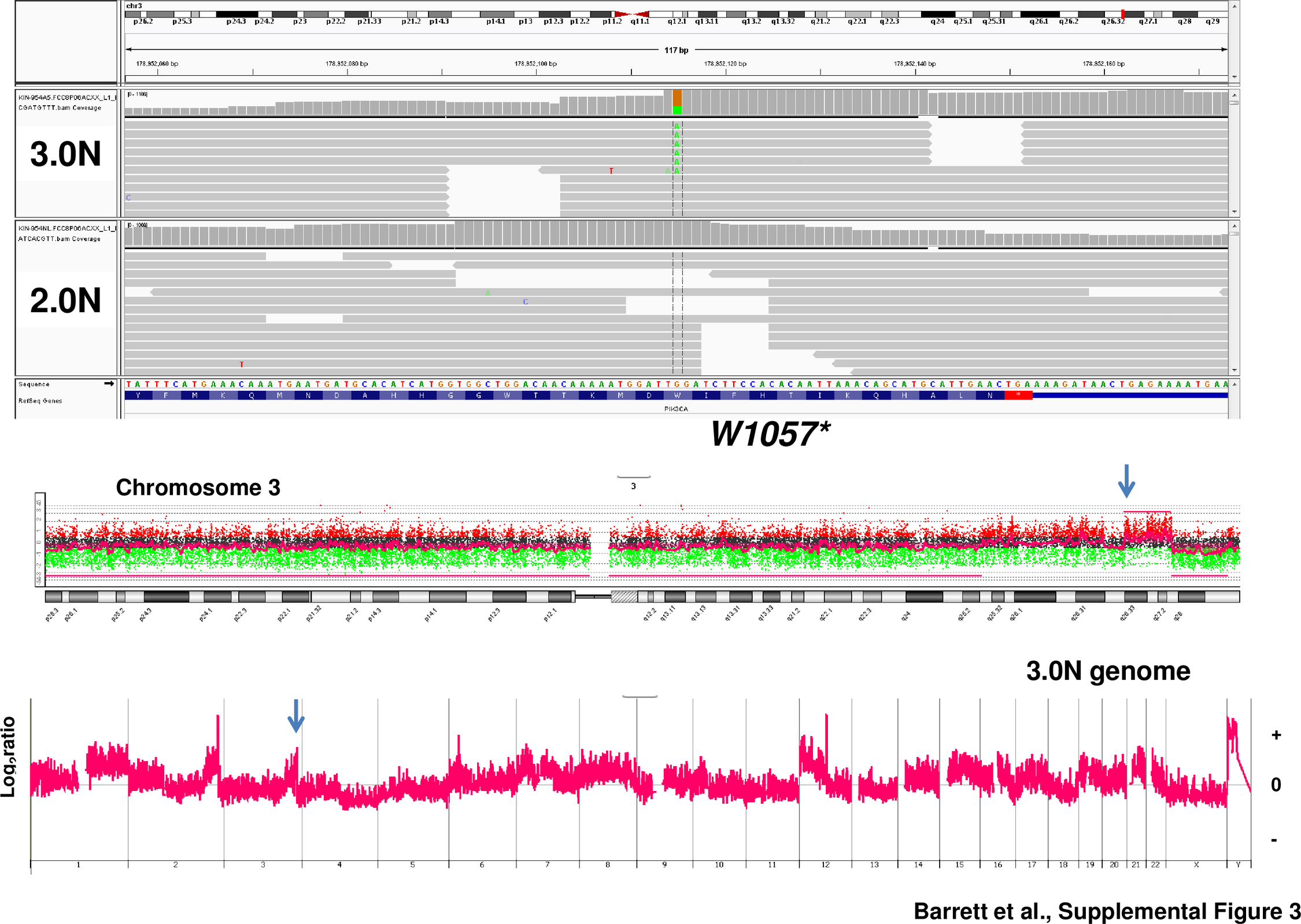

Supplement: S3 Fig — Whole genome (bottom panel) and chromosome 3 (middle panel) CNV profiles. The red shaded areas denote ADM2 defined copy number aberrant intervals. The X and Y axes in the CGH plots represent chromosome and log2ratios. C) IGV view of somatic PIK3CA mutation (top panel). (TIF) [file pone.0213815.s003.tif]
